# Presenilin 1 E280A mutation induces dysfunctional astrocytic phenotype in menstrual stromal-derived astrocyte-like cells

**DOI:** 10.1007/s00018-025-05902-7

**Published:** 2025-10-28

**Authors:** Natalia Quiroz Correa, Miguel Mendivil-Perez, Carlos Velez-Pardo, Marlene Jimenez-Del-Rio, Q. Quiroz-Correa

**Affiliations:** 1 Neuroscience Research Group, Institute of Medical Investigations, Faculty of Medicine, University of Antioquia (UdeA), Calle 70 No. 52-21, and Calle 62 # 52- 59, Torre 1, Laboratorio 412, Medellín, Colombia; 2 Neuroscience Research Group, Faculty of Nursing, University of Antioquia (UdeA), Calle 70 No. 52-21, and Calle 62 # 52-59, Torre 1, Laboratorio 412, Medellín, Colombia

**Keywords:** Alzheimer, Amyloid-beta, Astrocytes, E280A, IL-6, Neuroinflammation, Mutation, Presenilin

## Abstract

Familial Alzheimer’s disease (FAD) is a progressive neurodegenerative disorder associated with loss of cholinergic neurons, intra- and extracellular accumulation of amyloid beta (iAβ, eAβ), hyperphosphorylated tau, and neuroinflammation i.e., reactive glia. The precise contribution of astrocytes to neurodegeneration in FAD is still incompletely understood. Therefore, there is a need for a reliable and simple in vitro cell culture model of neuroinflammation. The aim of the present investigation was to determine the pathophysiological behavior of astrocyte-like cells (ALCs) derived from wild-type (WT) and PSEN1 E280A menstrual stromal cells (MenSCs). We found that WT and PSEN1 E280A MenSCs displayed similar cellular mesenchymal lineage markers and similar capacity to differentiate into mesenchymal lineage osteocytes, adipocytes, and chondrocytes. In addition, WT and mutant ALCs exhibited similar percentages of astrocyte lineage markers such as GFAP+ (~ 90%) and S100β+ (70%). However, compared to WT ALCs, PSEN1 E280A ALCs (i) showed a complete lack of response to Glu-induced Ca^2+^ influx; (ii) presented a high percentage of iAβ-positive cells (+ 3900% increase); (iii) exhibited a markedly dysfunctional phagocytosis activity (-70% reduction); (iv) revealed a dysregulation of apoptotic body clearance (-64% Lysotracker+/PI + cells); (v) showed abnormal accumulation of autophagosomes (+ 543% LC3-II + cells); (vi) revealed an increased TNF-α-induced activation of NF-κB (+ 37% MFI increase); (vii) an increased secretion of IL-6 (+ 160%), and (viii) secreted high amounts of Aβ42 (extracellular Aβ42, ~ 57 pg/mL). Notably, there was no change in TREM2 expression, mitochondrial membrane potential (ΔΨm, 100%), or induced cleaved caspase 3 (CC3, 0%) in PSEN1 E280A and WT ALCs. These findings highlight the importance of MenSCs-derived ALCs as a promising model to study the pathophysiological basis of FAD.

**Graphical abstract:**

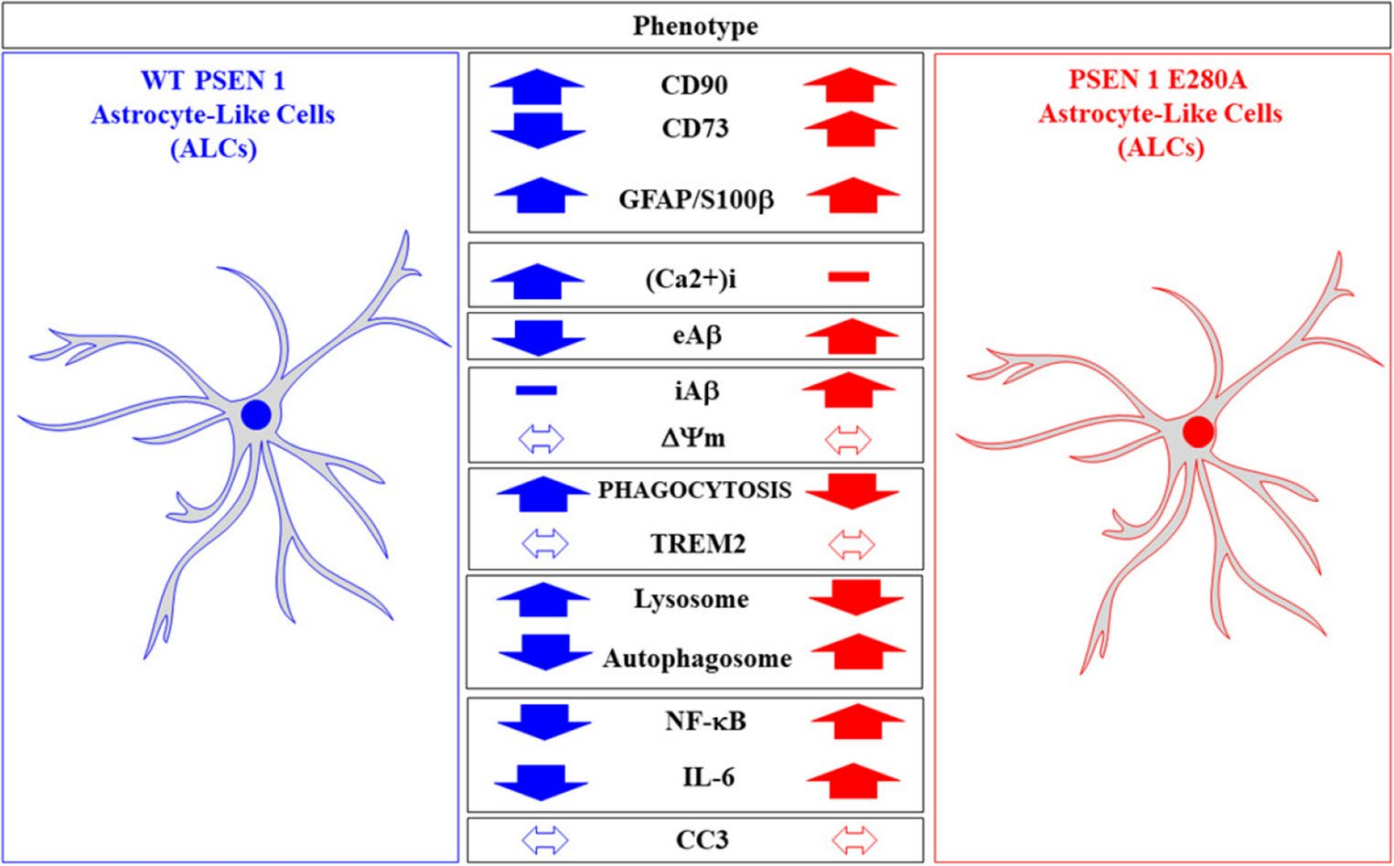

## Introduction

Alzheimer’s disease (AD) is an insidious chronic neurological disorder characterized by memory loss [1] due to severe loss of cholinergic neurons in the nucleus basalis of Meynert (cholinergic group Ch4) and medial septal nuclei (Ch1), which provide cholinergic innervation to the temporal pole and superior temporal cortex and hippocampus, respectively [2]; the presence of intracellular accumulations of amyloid-beta (iAβ), hyperphosphorylated tau (p-Tau)-containing neurofibrillary tangles and extracellular amyloid-β (eAβ)-containing plaques; and astroglia reactive cells, among other pathological markers [3, 4]. AD can occur either in a familial [5] or sporadic [6] manner. While sporadic AD is due to a complex combination of genes, environmental factors, or lifestyle [7], familial AD (FAD) is caused by a mutation in one of three genes known as the amyloid precursor protein (APP), presenilin 1 (PSEN1), and presenilin 2 (PSEN2) genes, which account for at least 103, 350, and 80 mutations, respectively (https://www.alzforum.org/mutations; accessed January 2025). Metabolically, APP can be metabolized by either the amyloid pathway or the non-amyloid pathway. While the non-amyloid pathway generates three fragments, such as soluble APP-α, p3, and amyloid intracellular domain (AICD), via α-secretase and γ-secretase cleaves, in the amyloid pathway, the action of a β-secretase followed by a γ-secretase generates soluble APP-β, the classical Aβ peptide (Aβ1–42), and AICD [8]. Interestingly, PSEN1 is the catalytic subunit of the protease γ-secretase, which cleaves more than 90 type I transmembrane proteins, including APP, Notch, and TREM2 (triggering receptor expressed on myeloid cells 2) [9]. However, mutations in PSEN1 profoundly affect its catalytic activity, leading to the overproduction and accumulation of Aβ1–42 [10, 11]. It is widely accepted that Aβ1–42, thought to be primarily produced by neurons, can activate a sustained inflammatory response that ultimately drives microglia and astrocytes to take it up and clear it from the brain [12]. Accordingly, several studies have demonstrated reactive microglia and astrocytes in the vicinity of Aβ plaques in postmortem brains of Alzheimer’s patients (e.g [13, 14]). Thus, chronic neuroinflammation plays an important role in the progression of AD [15, 16].

Astrocytes represent a heterogeneous population of cells with diverse functional and morphological characteristics [17, 18]. Astrocytes express several markers, including glial fibrillary acidic protein (GFAP) and calcium-binding protein S100β [19]. However, in response to eAβ1–42 and tau inflammatory stimuli, astrocytes undergo morphological, molecular, and functional remodeling, leading to reactive astrocytes [20]. Once they become reactive, astrocytes release several proinflammatory cytokines (e.g., interleukin-6, IL-6), triggering neuroinflammation [21]. Although astrocytic reactivity correlates with the severity of AD [22], the contribution of reactive astrocytes to the pathogenesis of AD remains elusive [23]. In addition to their role in neuroinflammation, astrocytes play several physiological roles involving the refinement of neuronal circuits and the coordination of synaptic networks related to the astrocyte-neuron axis [24]. They can also modulate synaptic transmission and plasticity, mainly by reuptake of glutamate from the synaptic cleft through their membrane glutamate transporters (e.g., GLT-1) and glutamate/aspartate transporters (e.g., GLAST) [25]. Interestingly, astrocytes can produce Aβ [26]. Given this wide range of functional properties and their importance in disrupting astrocyte-neuronal signaling [27], astrocytes have become therapeutic targets in AD [28, 29]. Despite these advances, the precise contribution of astrocytes to neurodegeneration in AD remains incomplete [30, 31]. Therefore, there is a need for a reliable and simple in vitro neuroinflammatory cell culture model [32, 33] to study the mechanisms behind astrocyte reactivity in AD.

Modeling of FAD using human induced pluripotent stem cells (hiPSCs), umbilical cord Wharton’s jelly mesenchymal stromal cells (WJ MSCs), and menstrual stromal cells (MenSC)-derived cholinergic-like cells (ChLNs) carrying the mutation in PSEN1 E280A (a glutamic acid to alanine change at codon 280, https://www.alzforum.org/mutations/psen1-e280a-paisa, accessed January 2025; [34]), which is 100% penetrant and affects a large population localized in Antioquia, Colombia [35], has provided an important biological tool to potentially recapitulate the neuropathology of the disease [36, 37]. Indeed, PSEN1 E280A ChLNs, but not wild-type (WT) ChLNs, exhibited increased intracellular Aβ (iAβ) fragments, eAβ42, and TAU phosphorylation (at residues Ser202/Thr205), recapitulating the molecular pathogenesis of AD. In addition, mutant ChLNs exhibited ACh-induced Ca²⁺ influx dysregulation compared to control ChLNs. Our group has also obtained WT astrocyte-like cells (ALCs, 59% GFAP+/S100β+) that respond to glutamate (Glu)-induced Ca^2+^ inward currents from MenSCs [38]. Furthermore, when WT MenSC-derived ALCs were challenged with PSEN1 E280A ChLN culture supernatant alone, IL-6 and activated nuclear factor-kappa B (NF-κB), indicators of neuroinflammation and reactive astrocytes, respectively, were significantly increased in ALCs compared to untreated or WT ChLN supernatant-treated ALCs [39]. However, the cellular consequences of astrocytes carrying the PSEN1 E280A mutation are unknown. To date, there is no in vitro model that allows recapitulation of the pathological features associated with potential alterations in astrocytic activity in patients carrying this specific mutation. Since MenSCs have been shown to be neuropathologically comparable to hiPSCs and WJ-MSCs [37], the present investigation aimed to determine the pathophysiological behavior of ALCs derived from WT and PSEN1 E280A MenSCs. To this end, WT and mutant MenSCs were first assessed for their ability to differentiate into the mesenchymal lineage and transdifferentiate into ALCs. In addition, WT and mutant ALCs were evaluated for their ability to express astrocytic markers. Furthermore, as ChLNs counterparts, ALCs were evaluated for their potential to endogenously accumulate iAβ fragments and cleaved caspase 3 (CC3). We also evaluated whether WT MenSCs-derived ALCs or mutant ALCs become reactive, i.e., show NF-κB-positive cells and secrete IL-6, when challenged with the pro-inflammatory cytokine TNF-α. We found that although WT and PSEN1 E280A MenSCs showed no differences in their ability to differentiate into mesenchymal lineage osteocytes, adipocytes, chondrocytes, and expressed similar percentages of astrocyte lineage markers such as GFAP+/S100β+, mutant ALCs (i) showed a complete lack of response to Glu-induced Ca^2+^ influx; (ii) presented a high percentage of iAβ-positive cells; (iii) exhibited a markedly dysfunctional phagocytosis activity according to E. coli fluorescence bioparticles^®^ phagocytosis assay; (iv) exhibited low apoptotic body phagocytosis associated with dysregulation of the phagolysosome/autophagolysosome formation process; (v) revealed high TNF-α-induced activation of NF-κB; and (vi) high secretion of IL-6. Remarkably, PSEN1 E280A ALCs do not alter ΔΨm or TREM2 expression or induce CC3. These findings highlight the importance of MenSCs-derived ALCs as a promising model to study the pathophysiological basis of FAD.

## Materials and methods

### Cells

Menstrual blood samples were collected from a healthy female and a female carrier of the PSEN1 E280A mutation at the ages of 29 years (Tissue Bank Code, TBC# MSC-MB0001; APOE*3/*3) and 20 years (TBC# MSC-MB0002; APOE*3/*4), respectively, according to [38]. Human astrocytes^®^ from ScienCell Research Laboratories (Cat No. 1800; 1610 Faraday Ave, Carlsbad, CA 92008,) isolated from the human brain (cerebral cortex), were used as a control and cultured according to the supplier’s recommendations. The APOE genotype was performed by LIME laboratory, Medellin-Colombia.

### Immuno-phenotypic characterization

Standard flow cytometry techniques were used to determine cell surface epitope profiles (CD105, CD90, CD45, CD73, CD11b, and CD34) in WT PSEN1 and PSEN1 E280A mutant cells. Briefly, cells were incubated with saturating concentrations (1:500) of mouse monoclonal antibodies conjugated to human CD105-phycoerythrin (PE, Cat. No. E-AB-F1310D), CD90-fluorescein isothiocyanate (FITC, Cat. No. E-AB-F1167C), CD45-PE (Cat. No. E-AB-F1137D), CD73-APC (Cat. No. E-AB-F1242E), CD11b-FITC (Cat. No. E-AB-F1146C), and CD34-APC (Cat. No. E-AB-F1143E). All antibodies were purchased from Elabscience (Houston, Texas, USA). Cells were incubated for 1 h at 4 °C. Prior to antibody labeling, cells were preincubated with 5% fetal bovine serum (FBS) for 10 min to block non-specific binding. Cell suspensions were washed and resuspended in PBS for analysis on an LSR-Fortessa (BD Biosciences). Ten thousand events were acquired, and acquisition analysis was performed using FlowJo 7.6.2. Positive staining was defined as fluorescence emission that exceeded the level of more than 99% of the cells in the population stained with the corresponding negative controls.

## Cell differentiation

### Osteogenic differentiation

Osteogenic differentiation was performed according to [40] with minor modifications. Briefly, WT and mutant MenSCs at passages 4–7 were plated at a density of 10,000 cells per cm² in 12-well plates in regular culture medium. After 72 h, the culture medium was replaced with osteogenic differentiation medium containing high-glucose DMEM (Sigma), 10% FBS, 1 µM dexamethasone (Alfa Aesar, cat # A17590), 250 µM sodium ascorbate (Sigma, cat # A4034), and 10 mM β-glycerophosphate (Alfa Aesar, cat # L03425). Medium was changed every 3–4 days. Control cells were maintained in regular culture medium (RCm). After 20 days of induction, cells were fixed in 4% FA and incubated with mouse anti-human osteocalcin monoclonal antibody (1:500; R&D, cat # MAB1419), followed by incubation with anti-mouse DyLight™ 594 secondary antibody (1:500) and 1 µM Hoechst 33,342 (Life Technologies).

### Adipogenic differentiation

Adipogenic differentiation was performed according to [40] with minor modifications. Briefly, WT and mutant MenSCs at passages 4–7 were plated at a density of 20,000 cells per cm² in a 12-well plate in regular culture medium. At 90–100% confluence, the culture medium was replaced with adipogenic induction medium containing high glucose DMEM, 10% FBS, 0.5 mM 3-isobutyl-1-methylxanthine (Sigma, cat # I5879), 100 µM indomethacin (Sigma, cat # I7378), 0.1 µM dexamethasone, and 10 µg/mL insulin. Control cells were maintained in regular culture medium. After 20 days of induction, cells were fixed in 4% FA and incubated with goat anti-mouse fatty acid-binding protein 4 (FABP4) antigen affinity-purified polyclonal antibody (1:500; R&D, cat# AF3150), followed by incubation with anti-goat DyLight™ 594 secondary antibody (1:500) and 1 µM Hoechst 33,342 (Life Technologies).

### Chondrogenic differentiation

Chondrogenic differentiation was performed according to [41] with minor modifications. Briefly, 5 × 10^4^ WT and mutant MenSCs were aggregated in microwell plates and then supplemented with chondrogenic medium containing high-glucose DMEM, 10% FBS, 10 µg/mL TGF-β3, 0.1 µmol/L dexamethasone, 50 µmol/L vitamin C, and 6.25 mg/mL insulin. The medium was changed every 3–4 days. Control cells were maintained in regular culture medium. After 20 days of induction, cells were fixed in 4% FA and incubated with goat anti-human aggrecan antigen affinity-purified polyclonal antibody (1:500; R&D, cat# 967800), followed by incubation with anti-goat DyLight™ 594 secondary antibody (1:500) and 1 µM Hoechst 33,342 (Life Technologies).

### Astrocyte-Like cells (ALCs) differentiation

Astrocyte differentiation was performed as described in [38]. Briefly, WT and PSEN1 E280A MenSCs were seeded at 1 × 10^4^ cells per cm^2^ in 25 cm^2^ culture flasks in regular culture medium (RCm, DMEM low-glucose media (Sigma Cat. N° D6046) supplemented with 10% FBS) until they reached 40% confluence. The medium was then changed, and the cells were incubated for 7 days in either DMEM low-glucose media supplemented with 2% FBS (minimal culture medium, MCm) or Astrocyte Medium^®^ (GIBCO^®^, Cat. N° A1261301).

### Immunofluorescence analysis

For the analysis of astrocytic and inflammation-related markers, cells treated under different conditions were fixed with 2% paraformaldehyde for 20 min, followed by Triton X-100 (0.1%) permeabilization and 5% fetal bovine serum (FBS) blocking. Cells were then incubated overnight with primary antibodies against glial fibrillary acidic protein (GFAP 1:200, Cat. N° ab16997, Abcam), S100 calcium-binding protein B (S100β, 1:500, Biolegend, Cat. N° 676604), amyloid β (amyloid βA4, clone 1E8 (amino terminal) antibody, 1:200, Millipore, Cat. N° MABN639), triggering receptor expressed on myeloid cells 2 (TREM2, 1:500, Thermo Fisher Scientific, cat. N° 702886), and nuclear factor kappa B (NF-κB/p65, 1:500, Thermo Fisher Scientific, cat. N° PA5-16545). After thorough rinsing, the cells were incubated with secondary fluorescent antibodies (Alexa Fluor 488 anti-rabbit, cat. N° A21206, and DyLight 594 horse anti-mouse, cat. N° DI 3088) at 1:500. Cell nuclei were stained with 1 µM Hoechst 33,342 (Life Technologies). Fluorescence microscopy images were captured using a Zeiss Axio Vert.A1 fluorescence microscope equipped with a Zeiss AxioCam Cm1 (Zeiss Wöhlk-Contact-Linsfluoreen, Gmbh, Schoenkirchen, Germany).

### Flow cytometry (FC) analysis of Astrocytic- and inflammation-related markers

Cells were detached with 0.25% trypsin-EDTA 1 mM and fixed in suspension with cold ethanol (−20 °C). After washing, cells were incubated with GFAP, S100β, amyloid β, TREM2, and NF-κB/p65 primary antibodies (1:200) overnight at 4 °C. Cell suspensions were washed and incubated with Alexa Fluor 488 Donkey anti-rabbit and DyLight 594 donkey anti-mouse antibodies (1:500). Finally, the cells were washed and resuspended in PBS for analysis on an LSR-Fortessa (BD Biosciences). Ten thousand events were acquired and acquisition analysis was performed using FlowJo 7.6.2 data analysis software. Positive staining was defined as fluorescence emission exceeding the level of the population stained with the negative control.

### Intracellular calcium imaging

Changes in intracellular calcium (Ca^2+^) concentration induced by glutamatergic stimulation were assessed according to [42] with minor modifications. The fluorescent dye Fluo-3 (Fluo-3 AM; Thermo Fisher Scientific, cat: F1242) was used for the measurement. The dye was dissolved in DMSO (1 mM) to form a stock solution. Before experiments, the stock solution was diluted in neuronal buffer solution (NBS buffer: 137 mM NaCl, 5 mM KCl, 2.5 mM CaCl₂, 1 mM MgCl₂, pH 7.3, and 22 mM glucose). The working concentration of dye was 2 µM. The WT and PSEN1 E280A MenSCs and ALCs were incubated with the dye containing NBS for 30 min at 37 °C and then washed five times. Intracellular Ca²⁺ transients were elicited by glutamate (Glu; 100 µM final concentration [43]). Measurements were performed using the 20x objective of the microscope. Several regions of interest (ROIs) were defined in the field of view of the camera. One of the ROIs was cell-free, and the fluorescence intensity measured here was considered as background fluorescence (F_bg_). The time dependence of the fluorescence emission was recorded, and the fluorescence intensities (hence the Ca^2+^ levels) were represented by pseudocolors. To calculate the changes in the average Ca²⁺-related fluorescence intensities, the Fbg value was determined from the cell-free ROI, and then the resting fluorescence intensities (F_rest_) of the cell-containing ROIs were obtained as the average of the points recorded during a consecutive period of 10 s before the addition of Ach or Glu. Peaks of fluorescence transients were found by calculating the average of six consecutive points and identifying the points that gave the highest average value (F_max_). The amplitudes of the Ca²⁺-related fluorescence transients were expressed relative to the resting fluorescence (ΔF/F) and calculated using the following formula: ΔF/F= (F_max_-F_rest_)/(F_rest_-F_bg_). ImageJ was used to calculate fluorescence intensities. The term fluorescence intensity was used as an indirect indicator of intracellular Ca^2+^ concentration.

### Phagocytosis assay

WT PSEN1 and E280A cells were incubated in astrocyte differentiation medium for 7 days. The cells were then treated with pHrodo^®^ red *E. coli* bioparticle conjugates (Thermo Fisher, Cat. No. P35361) at a final concentration of 10 ug/mL and analyzed by flow cytometry and fluorescence microscopy. Cell nuclei were stained with (0.5 µM) Hoechst 33,342 dye. Furthermore, the phagocytic capacity of ALCs was analyzed by incubating the cells with propidium iodide-labeled apoptotic bodies obtained from Jurkat cells or HEK293 cells treated with the apoptosis inducer TPEN [44]. The cells were then incubated with the cell-permeable green fluorescent dye that stains acidic compartments within a cell, LysoTracker^®^ Green DND-26 (20 nM, final concentration), for 20 min at RT in the dark (Cell Signaling Technology, cat. N°8783P). The cells were then washed twice with PBS. LysoTracker^®^ and propidium iodide (PI) fluorescence were analyzed by flow cytometry or by analysis of fluorescence microscopy images. Cell nuclei were stained with (1 µM) Hoechst 33,342 dye. To analyze Microtubule-Associated Protein 1 Light Chain 3-II (LC3-II) accumulation, ALCs that had been treated with unstained apoptotic bodies were detached using 0.25% trypsin-EDTA (1 mM) and then fixed in suspension using cold ethanol (−20 °C) or 2% paraformaldehyde for 20 min. This was followed by permeabilization using 0.1% Triton X-100 and blocking using 5% fetal bovine serum (FBS). The cells were then incubated overnight with a primary antibody against LC3-II (Cat. No. NB100-2220, Novus Biologicals, Englewood, CO, USA). After rinsing thoroughly, the cells were incubated with a secondary fluorescent antibody (DyLight 594 horse anti-mouse, catalog number DI3088) at a dilution of 1:500. For flow cytometry analysis, the cells were washed and resuspended in PBS and analyzed on an LSR-Fortessa (BD Biosciences). Ten thousand events were acquired and analyzed using FlowJo 7.6.2 data analysis software. Positive staining was defined as fluorescence emission exceeding the level of the population stained with the negative control. For fluorescence microscopy analysis, the cell nuclei were stained with 1 µM Hoechst 33,342 (Life Technologies), and images were captured using a Zeiss Axio Vert.A1 fluorescence microscope equipped with a Zeiss AxioCam MRm (Zeiss Wöhlk Contact Lenses GmbH, Schoeneckirchen, Germany).

### Analysis of caspase 3 activation and mitochondrial membrane potential (ΔΨm) by fluorescence microscopy

ALCs were incubated with the substrate for caspase-3 detection (Vybrant™ FLICA Caspase Apoptosis Assay Kits, Thermo Fisher Scientific, Cat. No. V35118) and with the passively diffusing and active mitochondria accumulating dye deep red MitoTracker^®^ compound (20 nM, final concentration) for 20 min at room temperature in the dark (Invitrogen, Cat. No. M22426). The cells were then washed twice with PBS. Caspase-3 and MitoTracker^®^ fluorescence intensities were determined by analysis of fluorescence microscopy images. The evaluation was repeated three times in independent experiments. Cell nuclei were stained with (0.5% µM) Hoechst 33,342 dye.

### Analysis of caspase-3 activation and mitochondrial membrane potential (ΔΨm) by flow cytometry

ALCs were incubated with MitoTracker^®^ (20 nM, final concentration) and the substrate for caspase-3 detection for 30 min at RT in the dark. Cells were analyzed using an LSR-Fortessa (BD Biosciences). The experiment was performed in triplicate in independent experiments, and 10,000 events were acquired for analysis. Quantitative data and figures were obtained using FlowJo 7.6.2 data analysis software. The evaluation was repeated three times in independent experiments, blinded to the experimenter and flow cytometer analyst.

### Interleukin-6 (IL-6) measurement

WT and PSEN1 E280A ALCs were challenged with TNF-α (Sino Biological, Cat. No. 10602-HNAE) at a final concentration of 100 ng/mL for 24 h. Cells were then fixed for NF-κB assessment, and IL-6 levels were measured in cell-free supernatants using the Cytometric Bead Array (CBA) of Human Inflammatory Cytokines (Cat. No. 551811; BD Biosciences) according to the manufacturer’s protocol. Data were collected using a CytoFLEX flow cytometer (Beckman Coulter).

### Measurement of Aβ 1–42 peptides in culture medium

The amount of secreted Aβ1–42 peptides was measured according to a previous report with minor modifications [45]. Briefly, wild-type (WT) and presenilin 1 (PSEN1) E280A mesenchymal stem cells (MenSCs) were left in RPMI medium or differentiated into adipocytes for seven days (see above). Then, 100 µL of the resulting supernatant was collected, and the levels of secreted Aβ1–42 peptides were determined using the Human Abeta 42 solid-phase sandwich ELISA (Cat. No. KHB3441, Invitrogen, Waltham, MA, USA), following the manufacturer’s instructions. This assessment was repeated three times in independent experiments, blind to the experimenter.

### Photomicrography and image analysis

Fluorescence microscopy photographs were taken using a Zeiss Axiovert A1 fluorescence microscope equipped with a Zeiss AxioCam MRm1 and Zeiss Wöhlk Contact Linsfluoreen (GmbH, Schönkirchen, Göttingen, Germany). The fluorescence images were analyzed using ImageJ software (http://imagej.nih.gov/ij/, accessed May 7, 2023). The figures were transformed into 8-bit images, and the background was subtracted. Cellular regions of interest (ROIs) were drawn around the nucleus for transcription factors and apoptosis effectors or over all cells for cytoplasmic probes. The fluorescence intensity was then determined by applying the same threshold to cells in the control and treatment conditions. Mean fluorescence intensity (MFI) was obtained by normalizing total fluorescence to the number of nuclei.

### Data analysis

In this experimental design, two vials of MenSCs (WT and PSEN1 E280A) were thawed and cultured, and the cell suspension was pipetted at a standardized cell density of 2 × 10^4^ cells/cm^2^ into different wells of a 24- or 6-well plate. Cells (i.e., the biological and observational units) [46] were randomized to wells by simple randomization (sampling without replacement method), and then wells (i.e., the experimental units) were randomized to treatments by a similar method. Experiments were performed on three independent occasions (*n* = 3), blinded to the experimenter and/or flow cytometer analyst. The data from the three replicates, i.e., independent experiments, were averaged, and a representative flow cytometry density plot or histogram from the three independent experiments was selected for illustration, whereas the bars in the quantification figures represent the mean ± SD, and the three black dots represent the data point of each experimental replicate. Based on the assumptions that the data from the experimental unit (i.e., well) conform to the independence of observations, that the dependent variable is normally distributed in each treatment group (Shapiro-Wilk test), and that there is homogeneity of variances (Levene’s test), statistical significance was determined by Student’s t-test or one-way analysis of variance (ANOVA) followed by Tukey’s post hoc comparison calculated with GraphPad Prism 5.0 software. Differences between groups were considered significant only when the p-value was 0.05 (*), 0.01 (**), and 0.001 (***). All data are expressed as mean ± S.D.

## Results

### Wild-type and PSEN1 E280A menstrual stromal Cells (MenSCs) share similar mesenchymal lineage surface markers and mesodermal differentiation capacity.

**Fig. 1:**
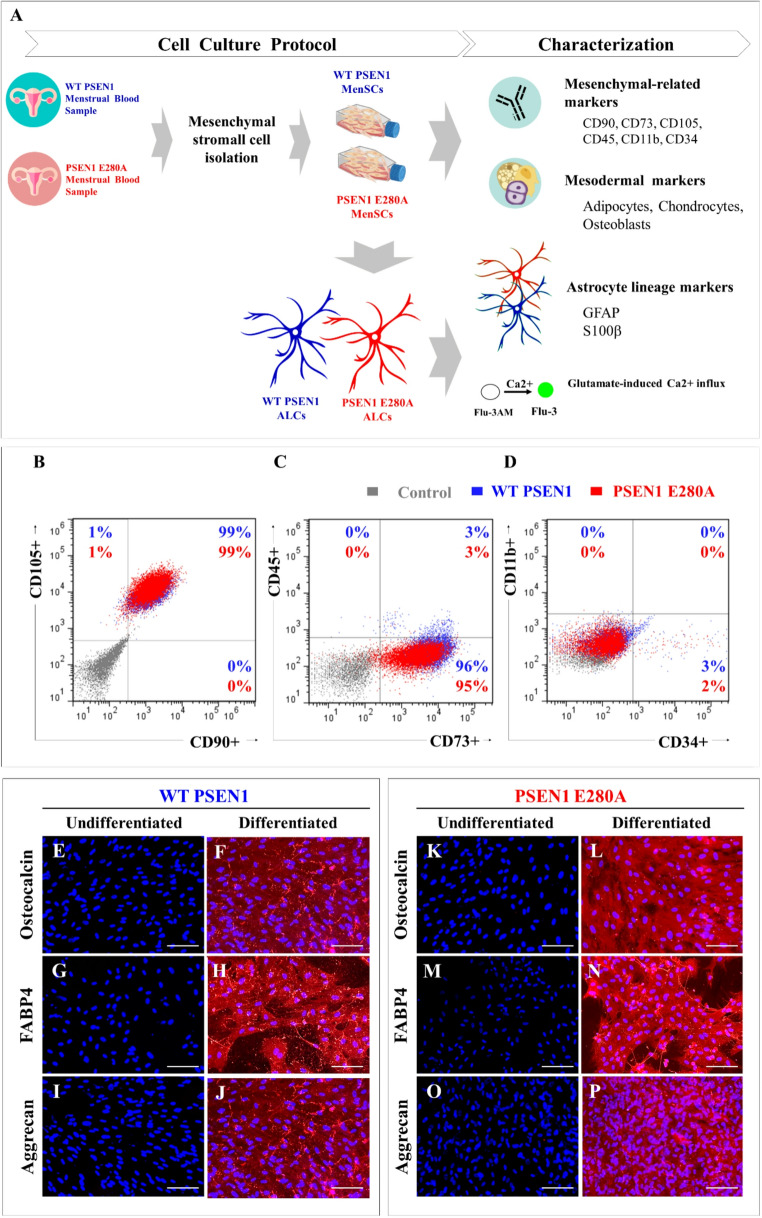
Characterization of WT PSEN1 and PSEN1 E280A MenSCs.(**A**) Menstrual blood sample from a healthy woman (WT PSEN1) and carrier of presenilin 1 at codon E280A (PSEN1 E280A) were used to isolate mesenchymal stromal cells (MenSCs) and differentiated into mesenchymal lineages. These cells were then characterized to express mesenchymal-related markers and mesodermal-differentiated lineage markers. (**B**-**D**) Flow cytometry analyses showing the percentage of double-positive markers in MSCs with antibodies anti-CD105 and -CD90 (B), anti-CD45 and -CD73 (**C**), anti-CD11b and -CD34 (**D**) surface antigens. (**E**-**P**) MSCs were cultured without or with specific differentiation medium. Then, cells were stained with primary antibodies against osteocalcin (**E**-**L**), FABP4 (**G**-**N**) and aggrecan (**I**-**P**). The nuclei were stained with Hoechst 33,342 (blue fluorescence). Image magnification 20x

It has been shown that naïve MenSCs express mesenchymal surface markers and are capable of differentiating into mesodermal lineages such as adipocytes, chondrocytes, and osteoblasts [38]. Therefore, we first sought to determine whether the PSEN1 E280A mutation affects the ability of MenSCs to express mesenchymal surface proteins and to differentiate into mesodermal lineages according to the experimental plan (Fig. 1A). Accordingly, flow cytometry analysis shows that both naïve and mutant ALCs did not express pan-hematopoietic cell marker CD45 (Fig. 1C), macrophage/microglia CD11b (Fig. 1D), or hematopoietic stem cell marker CD34 (Fig. 1D), but expressed mesenchymal lineage markers CD90, CD105 (Fig. 1B), and CD73 (Fig. 1C). Furthermore, there was no statistical difference between WT and PSEN1 E280A MenSCs expressing these markers (Fig. 1B and C). Immunofluorescence microscopy analysis shows that WT and PSEN1 E280A MenSCs cultured in regular culture medium (RCm) showed no detectable lineage markers, representing an undifferentiated stage of cells (Fig. 1E, G, I, K and M, and 1O), whereas WT and mutant MenSCs cultured in osteogenic, adipogenic, or chondrogenic induction medium differentiated into osteoblasts (Fig. 1F and L), adipocytes (Fig. 1H and N), and chondrocytes (Fig. 1J and P), respectively, based on osteocalcin-, fatty acid binding protein 4 (FABP4)-, and aggrecan-positive cells (reflected as red fluorescence).

### WT and PSEN1 E280A menstrual stromal cells (MenSCs)-derived astrocyte-like cells (ALCs) show similar levels of expression of the astrocytic markers GFAP and S100β, but PSEN1 E280A ALCs were unresponsive to glutamate-induced Ca^2+^ influx

.

**Fig. 2:**
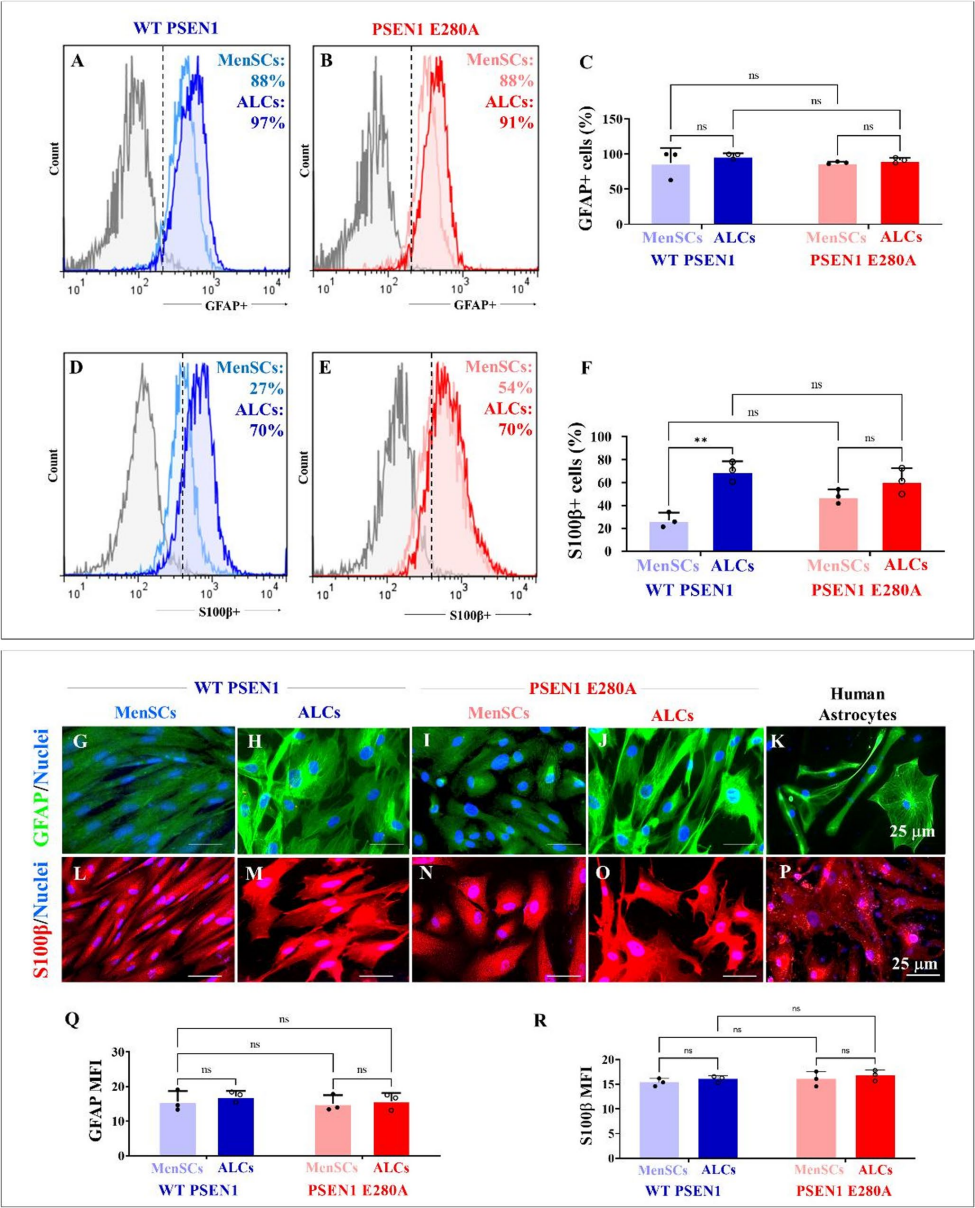
Astrocyte-associated markers expression in wild type (WT) and PSEN1 E280A astrocyte-like cells (ALCs). WT MenSCs (blue fluorescence) and PSEN1 E280A MenSCs (red fluorescence) were cultured in either regular culture medium or astrocyte medium. Representative flow cytometry histograms showing the percentage of GFAP (**A**, **B**) and percentage of GFAP-positive cells (**C**). Representative flow cytometry histograms showing the percentage of S100β-positive cells (**D**, **E**) and percentage of S100β-positive cells (**F**). Merge fluorescence microscopy images showing double stained with antibodies against GFAP (green fluorescence) and Hoechst 33,342 (blue fluorescence) in WT PSEN 1 MenSCs (**G**), WT PSEN1 ALCs (**H**), PSEN 1 E280A MenSCs (**I**), PSEN1 E280A ALCs (**J**), and human astrocyte (Control, **K**). Merge fluorescence microscopy images showing double stained with antibodies against S100β (red fluorescence) and Hoechst 33,342 (blue fluorescence) in WT PSEN 1 MenSCs (**L**), WT PSEN1 ALCs (**M**), PSEN 1 E280A MenSCs (**N**), PSEN1 E280A ALCs (**O**), and human astrocyte (Control, **P**). Quantification of S100β mean fluorescence intensity (**Q**). Quantification of S100β mean fluorescence intensity (**R**). The figures represent 1 out of 3 independent experiments. Data are expressed as the mean ± SD; **p < 0.01, ns = not significant. Image magnification 20x

The above observations prompted us to evaluate whether PSEN1 mutation alters the expression of astrocytic markers GFAP/S100β and whether mutant ALCs are functionally responsive to glutamate (Glu)-induced Ca^2+^ influx. As shown in Fig. 2, WT and PSEN1 E280A MenSCs and ALCs expressed similar levels of the GFAP marker by flow cytometry (Fig. 2A-C). Furthermore, flow cytometry analysis shows that although the expression of S100β was higher in PSEN1 E280A MenSCs than in WT MenSCs (Fig. 2D and E), its expression was not significantly different between WT ALCs and PSEN1 E280A ALCs (Fig. 2F). However, immunocytochemical analysis shows no statistical difference between GFAP and S100β in WT (Fig. 2G and L) and mutant MenSCs (Fig. 2I, N, Q and R) and ALCs (Fig. 2H, M, J, O, Q and R). As expected, human astrocytes (cerebral cortex) included as control expressed the astrocyte lineage markers GFAP-positive (Fig. 2K) and S100β−positive (Fig. 2P).

**Fig. 3:**
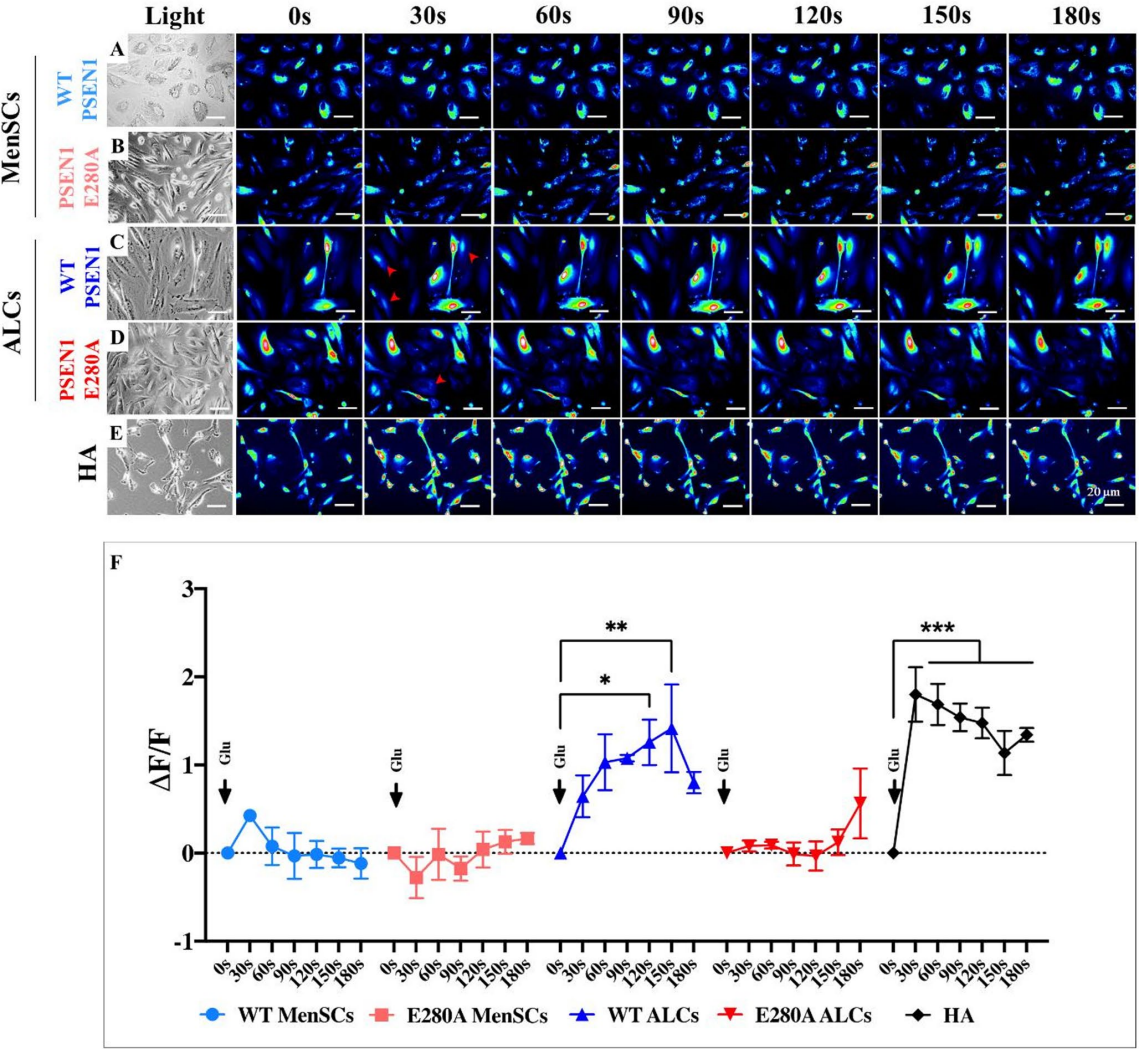
PSEN1 E280A ALCs are irresponsive to Glutamate-induced transient Ca^2+^ ion influx. concentration. Time-lapse images (0, 30, 60, 90, 120, 150 and 180 s) of Ca2+ fluorescence in WT MenSCs (**A**), PSEN1 E280A MenSCs (**B**), WT ALCs (**C**), PSEN1 E280A ALCs (**D**), and human astrocytes (**E**) as a response to glutamate (Glu) treatment. Glutamate was puffed into the culture at 0 s (black arrow). Activated nuclei are shown in red arrows. Then, Ca2+ fluorescence of cells was monitored at indicated times (n = 10 cells imaged, N = 3 dishes). Color contrast indicates fluorescence intensity: dark blue < light blue < green < yellow < red. Normalized mean fluorescence signal (ΔF/F) over time, indicating temporal cytoplasmic Ca2+ elevation in response to glutamate treatment (**F**). Red arrows in C and D show typical irresponsive ACLs to Glu. One-way ANOVA, post hoc test Bonferroni. Data are presented as mean ± SD; *p < 0.05; **p < 0.01; ***p < 0.001. Image magnification 20x

Previous studies have shown that glutamate (Glu) induces Ca^2+^ influx in astrocytes [47, 48]. Therefore, we tested whether the E280A mutation affects the response of astrocytes to Glu. To this end, WT/mutant MenSCs and WT/mutant ALCs were exposed to Glu. Figure 3 shows that Glu did not induce Ca^2+^ transient influx in WT MenSCs (Fig. 3A), mutant MenSCs (Fig. 3B), or PSEN1 E280A ALCs (Fig. 3D), but Glu induced Ca^2+^ influx in WT ALCs (Fig. 3C) and HA (Fig. 3E). Indeed, the maximal fluorescence change (ΔF/F) in WT ALCs was 1.7 ± 0.05-fold and in HA was 1.14 ± 0.25-fold after 150 s of Glu exposure (Fig. 3F, *n* = 30 ALCs imaged, *N* = 3 dishes).

### PSEN1 E280A menstrual stromal cells (MenSCs) and astrocyte-like cells (ALCs) produce intracellular iAβ fragments, but neither show mitochondrial membrane potential (ΔΨm) nor cleaved caspase 3 (CC3) in mutant cells

**Fig. 4:**
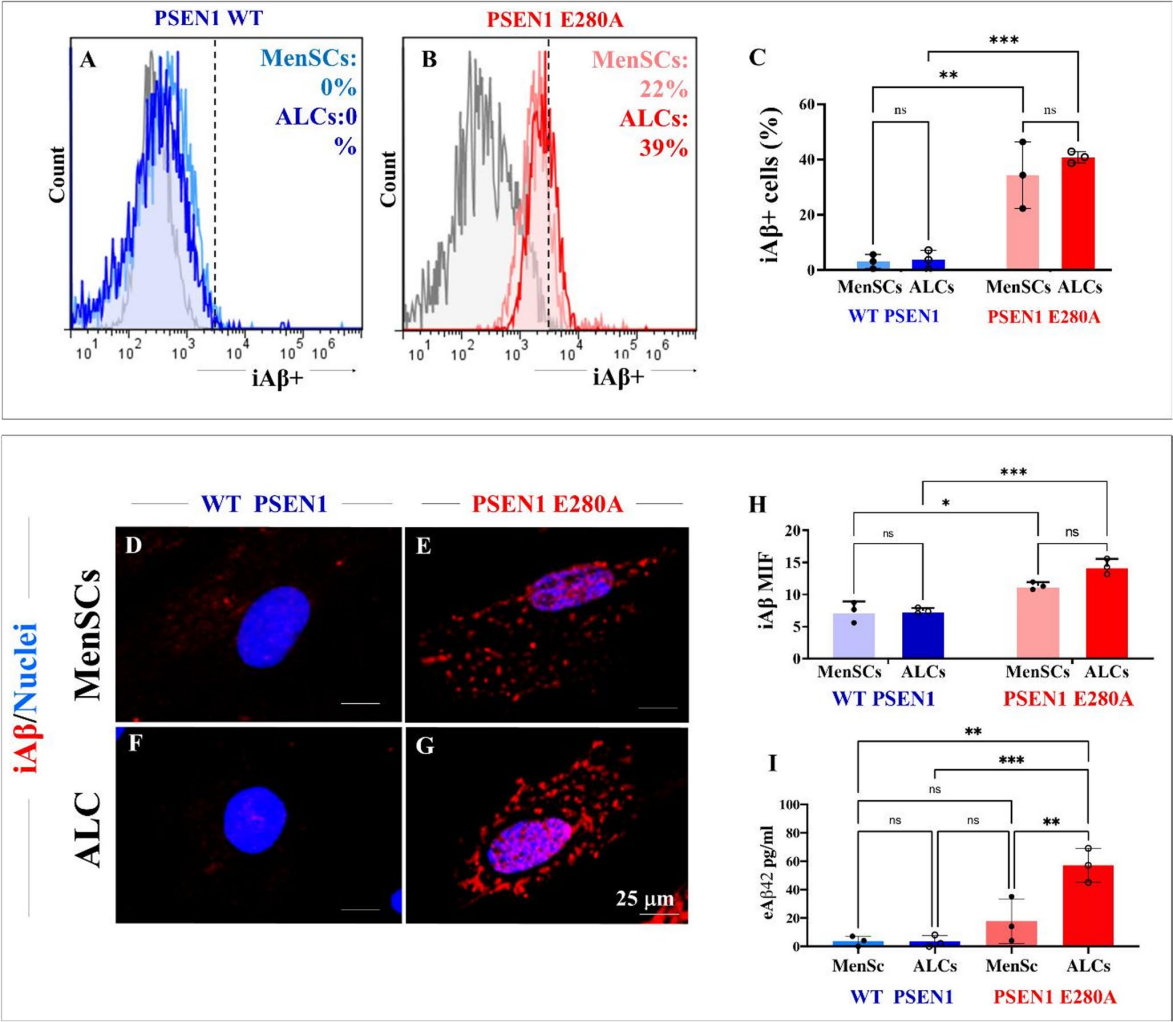
PSEN1 E280A cells show accumulation of intracellular Aβ (iAβ). WT MenSCs (blue fluorescence) and PSEN1 E280A MenSCs (red fluorescence) were cultured in either regular culture medium or astrocyte medium. Representative flow cytometry histograms showing the percentage of iAβ (**A**, **B**) and percentage of iAβ-positive cells (**C**). Merge fluorescence microscopy images showing Double stained with 1E8 (amino terminal) antibody against iAβ (red fluorescence) and Hoechst 33,342 (blue fluorescence) in WT PSEN 1 MenSCs (**D**), PSEN 1 E280A MenSCs (**E**), WT PSEN1 ALCs (**F**), and PSEN1 E280A ALCs (**G**). Quantification of iAβ mean fluorescence intensity (**H**). Quantification of eAβ42 in the culture supernatants (**I**). The figures represent 1 out of 3 independent experiments. Data are expressed as the mean ± SD; *p < 0.05; **<0.01; ***p < 0.001; ns = not significant. Anti-Aβ antibody = amyloid βA4, clone 1E8 (amino terminal) antibody. Image magnification 20x

Previously, it has been shown that MenSCs-derived ChLNs exhibit evident intracellular accumulation of Aβ fragments, loss of ΔΨm, and high CC3-positive cells [37, 49]. Therefore, we investigated whether PSEN1 E280A induces overproduction of intracellular Aβ, changes in ΔΨ_m_, or induction of the apoptosis marker CC3 in quiescent ALCs. Flow cytometry shows that WT MenSCs and ALCs do not produce detectable iAβ (Fig. 4A and C), whereas PSEN1 E280A MenSCs and ALCs increased the generation of iAβ by 2200% and 3900%, respectively (Fig. 4B and C). Similar observations were obtained by immunofluorescence microscopy analysis (Fig. 4D-H).

#### PSEN1 E280A astrocyte-like cells (ALCs) secrete high amounts extracellular Aβ (eAβ) into culture supernatant

To further characterize ALCs, we evaluated whether mutant astrocytes secrete extracellular Aβ. Figure 4I shows that WT MenSCs, WT ALCs, and PSEN1 E280A MenSCs secreted insignificant (~ 3.6 ± 3.3 pg/mL) or low amounts (~ 17.6 ± 15.0 pg/mL) of extracellular Aβ42 (eAβ42). However, PSEN1 E280A ALCs secreted three times more eAβ42 (~ 57 pg/mL) than PSEN1 E280A MenSCs did.

**Fig. 5:**
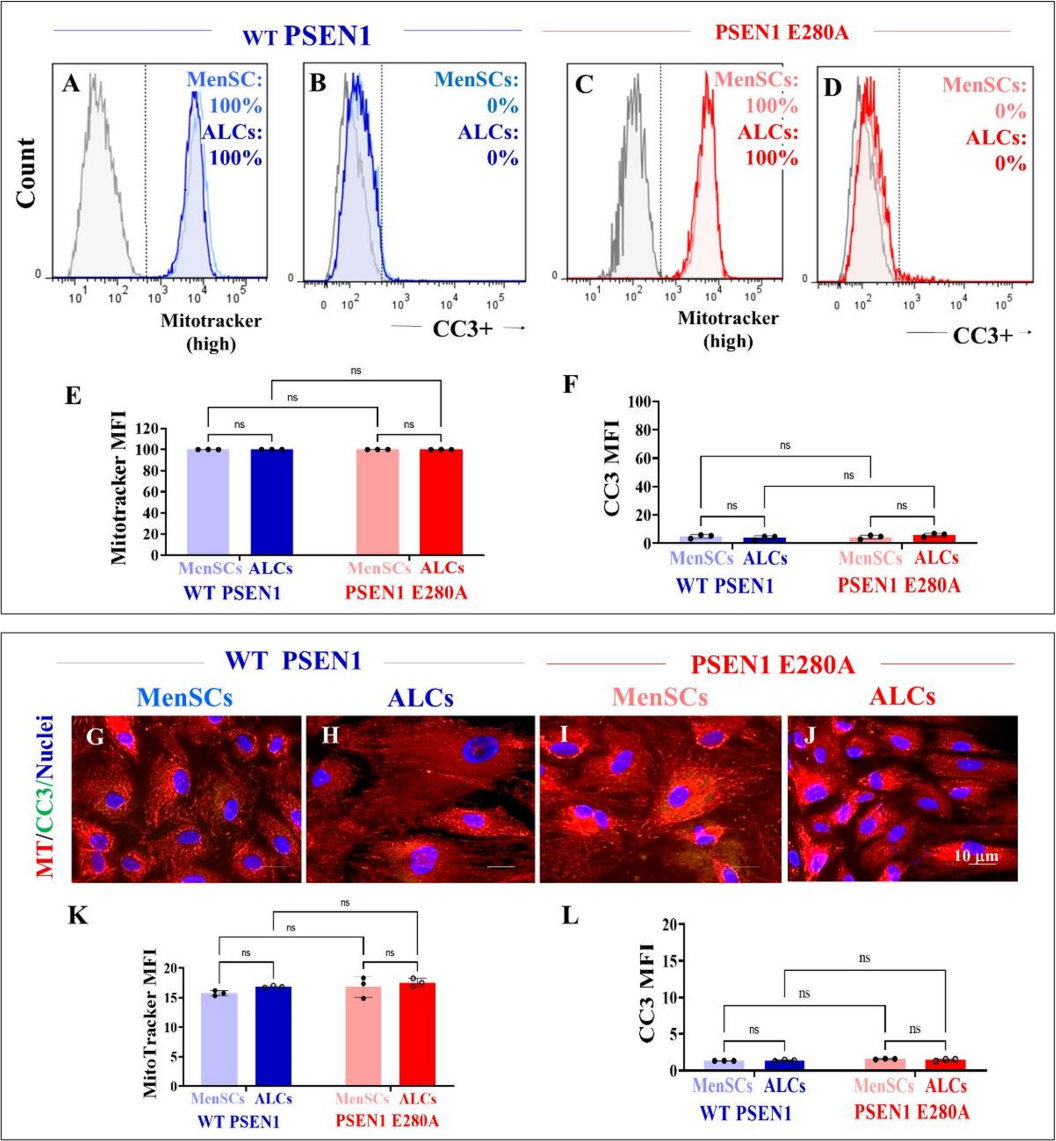
WT and PSEN1 E280A cells show similar levels mitochondrial membrane potential (ΔΨm) and caspase-3 activation. WT MenSCs, ALCs (blue fluorescence) and PSEN1 E280A MenSCs, ALCs (red fluorescence) were cultured in either regular culture medium or astrocyte medium. Representative flow cytometry histograms showing the percentage of MitoTracker (**A**, **B**) and percentage of cleaved caspase 3 (CC3)-positive cells (**C**, **D**). Representative flow cytometry histograms showing the percentage of MitoTracker-positive cells (**E**) and percentage of CC3-positive cells (**F**). Merge fluorescence microscopy images showing triple stained with antibodies against CC3 (green fluorescence), MitoTracker® (red fluorescence), and Hoechst 33,342 (blue fluorescence) in WT PSEN 1 MenSCs (**G**), WT PSEN1 ALCs (**H**), PSEN 1 E280A MenSCs (**I**), PSEN1 E280A ALCs (**J**). Quantification of MitoTracker® mean fluorescence intensity (**K**). Quantification of CC3 mean fluorescence intensity (**L**). The figures represent 1 out of 3 independent experiments. Data are expressed as the mean ± SD; ns = not significant. Image magnification 60x

Surprisingly, we found no changes in ΔΨ_m_ (Fig. 5A and B) or CC3-positive cells (Fig. 5C and D) in either WT or mutant MenSCs or ALCs (Figs. 5A-F). Similar results were obtained by immunofluorescence microscopy analysis (Figs. 5G-L).

### PSEN1 E280A ALCs, but not wild-type, show reduced phagocytic capacity and accumulation of autophagosomes

**Fig. 6:**
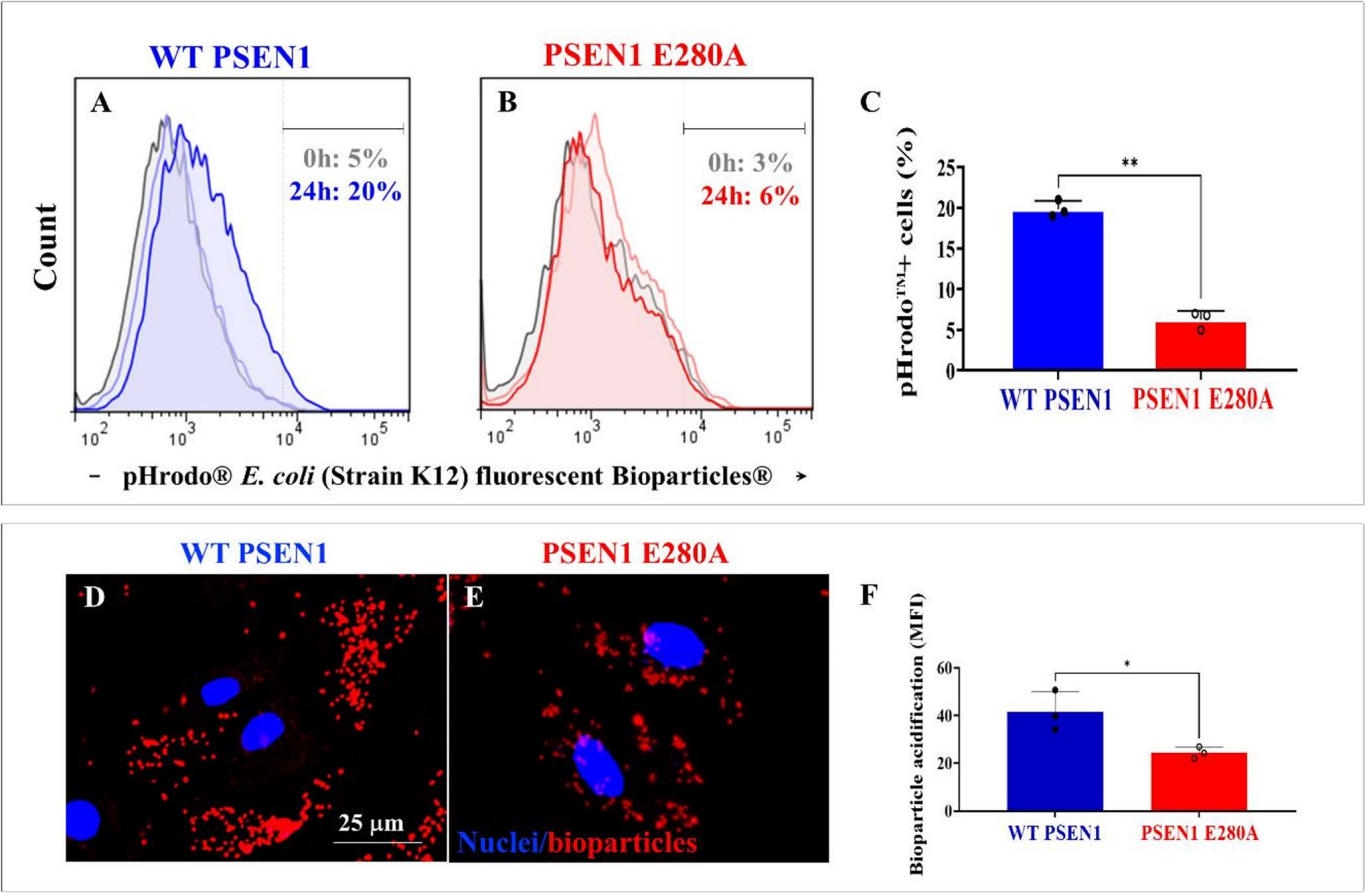
PSEN1 E280A ALCs show reduced phagocytic capacity of pHrodo *E. coli* fluorescent bioparticles. WT ALCs (blue fluorescence) and PSEN1 E280A ALCs (red fluorescence) were incubated with pHrodo E. coli fluorescent bioparticles for 24 h. Representative flow cytometry histograms showing the percentage of pHrodo bioparticles positive in WT (**A**) and PSEN1 E280A (**B**) ALCs. Percentage of pHrodo bioparticles positive cells (**C**). Representative microscopy images showing pHrodo fluorescent bioparticles (red fluorescence) in WT (**D**) and PSEN1 E280A (**E**) ALCs. The nuclei were stained with Hoechst 33,342 (blue fluorescence, D-E). pHrodo bioparticles fluorescence intensity quantification (**F**). Data are expressed as the mean ± SD; *p < 0.05; **p < 0.01. Image magnification 100x

Astrocytes are known to remove bacteria and dying or dead cells by phagocytosis to maintain a healthy CNS [50]. Therefore, we evaluated the phagocytic capacity of ALCs by exposing the cells to either pHrodo^®^ *E. coli* (strain K12) fluorescent Bioparticles^®^ or propidium iodine (PI)-stained apoptotic bodies and LysoTracker^®^ probe as described in the *Materials and Methods* section. Flow cytometry analysis shows that WT ALCs were able to phagocytose *E. coli* bioparticles by 20% after 24 h (Fig. 6A and C), i.e., a + 300% increase compared to baseline at 0 h (Fig. 6A), whereas PSEN1 E280A ALCs showed a significant impairment to phagocytose *E. coli* bioparticles by 6% phagocytic activity after 24 h (Fig. 6C and D), i.e., a −70% reduction compared to WT ALCs (Fig. 6C). Further analysis using pHrodo BioParticles conjugates, which are non-fluorescent outside the cell at neutral pH but fluoresce brightly in acidic pH environments such as those of phagosomes and lysosomes, shows that phagocytized bioparticles became more fluorescent due to the induction of acidic pH environments in ALCs, as represented by the red-point fluorescent dye, in WT ALCs (Fig. 6D and F) than in PSEN1 E280A ALCs (Fig. 6E and F).

**Fig. 7:**
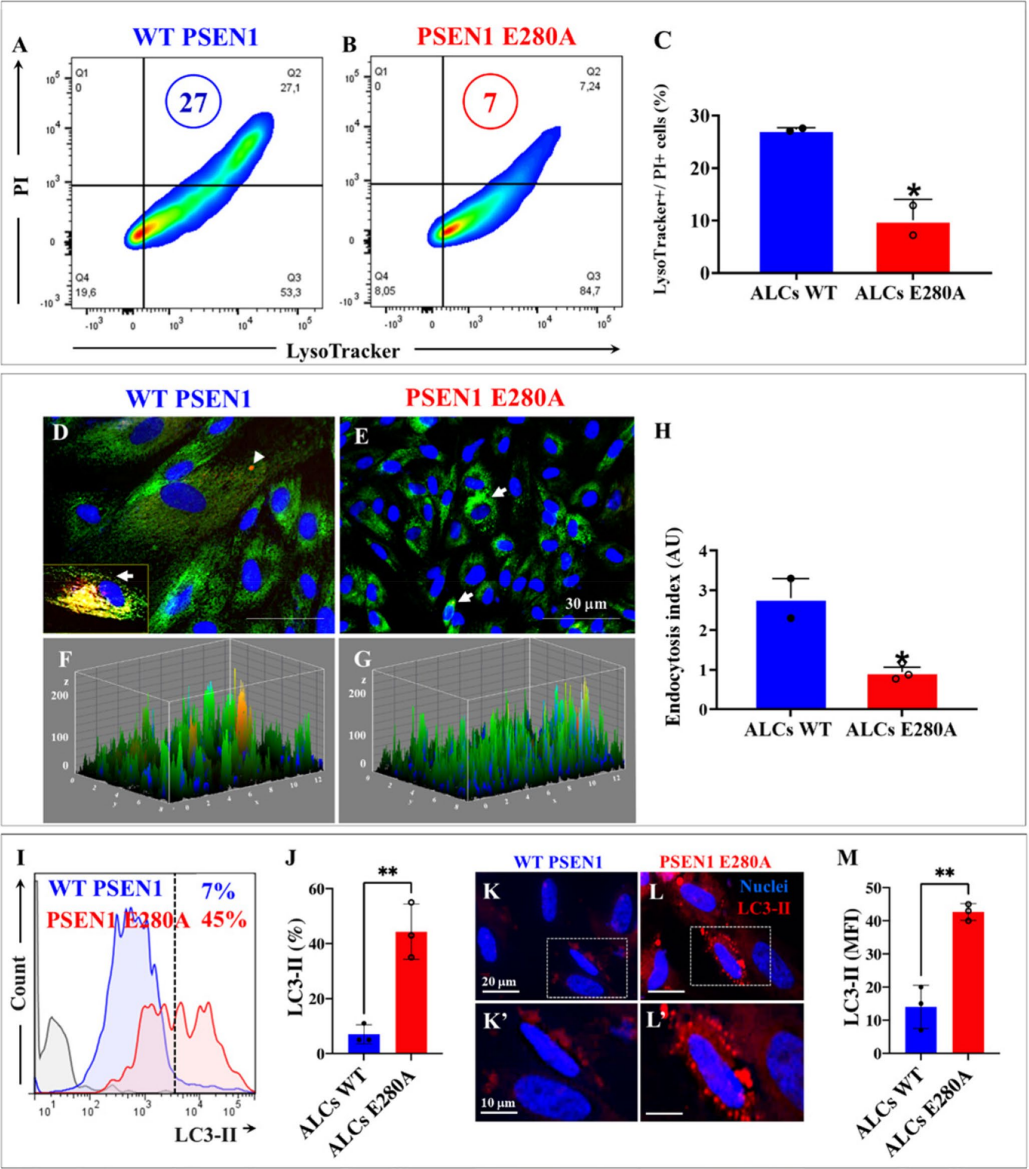
PSEN1 E280A ALCs show reduced phagocytic capacity of apoptotic bodies. WT PSEN1 and PSEN1 E280A ALCs were incubated with PI-labeled apoptotic bodies (previously prepared as described in Materials and methods section) for 30 min. Then, ALCs were incubated with the green fluorescent dye Lysotracker®. Representative density plot figures showing the PI/LysoTracker® double-positive population of WT ALCs (**A**) and PSEN1 E280A ALCs (**B**). Percentage of PI/LysoTracker® double-positive cells (**C**). Representative immunofluorescence microscopy of WT (**D**) and PSEN1 E280A (**E**) ALCs. The nuclei were stained with Hoechst 33342 (blue fluorescence, **D**-**E**). Fluorescent area representation of images obtained by immunofluorescence analysis (**F**, **G**). PI and LysoTracker® colocalization quantification of images obtained by immunofluorescence analysis, phagocytosis index (**H**). Representative flow cytometry histograms showing the percentage of accumulation of LC3-II positive cells in WT and PSEN1 E280A ALCs (**I**). Percentage of LC3-II (**J**). Representative immunofluorescence microscopy of WT (**K**, inset broken white line **K**’) and PSEN1 E280A (**L**, inset broken white line **L**’). ALCs. The nuclei were stained with Hoechst 33,342 (blue fluorescence, **K**, **L**). Quantification of LC3-II mean fluorescence intensity (M). Data are expressed as the mean ± SD; *p < 0.05; **p < 0.01. Image magnification 20x

To further validate the above observations, we exposed ALCs to PI-positive apoptotic bodies and evaluated their co-localization with lysosomes using the Lysotracker^®^ reagent as described in the *Materials and Methods* section. As shown in Fig. 7, the double-stained PI and Lysotracker value (Q2 in Fig. 7 A versus 7B) was higher in WT ALCs (Fig. 7A) than in PSEN1 E280A ALCs (Fig. 7B). Indeed, mutant ALCs decreased PI/Lysotracker^®^ dye by −74% compared to WT ALCs (Fig. 7C). Furthermore, fluorescence microscopy examination (Figs. 7D-G) reveals a significant impairment in the phagocytosis of PI-positive apoptotic bodies by PSEN1 E280A compared to WT ALCs according to the phagocytosis index analysis (Fig. 7H).

**Fig. 8:**
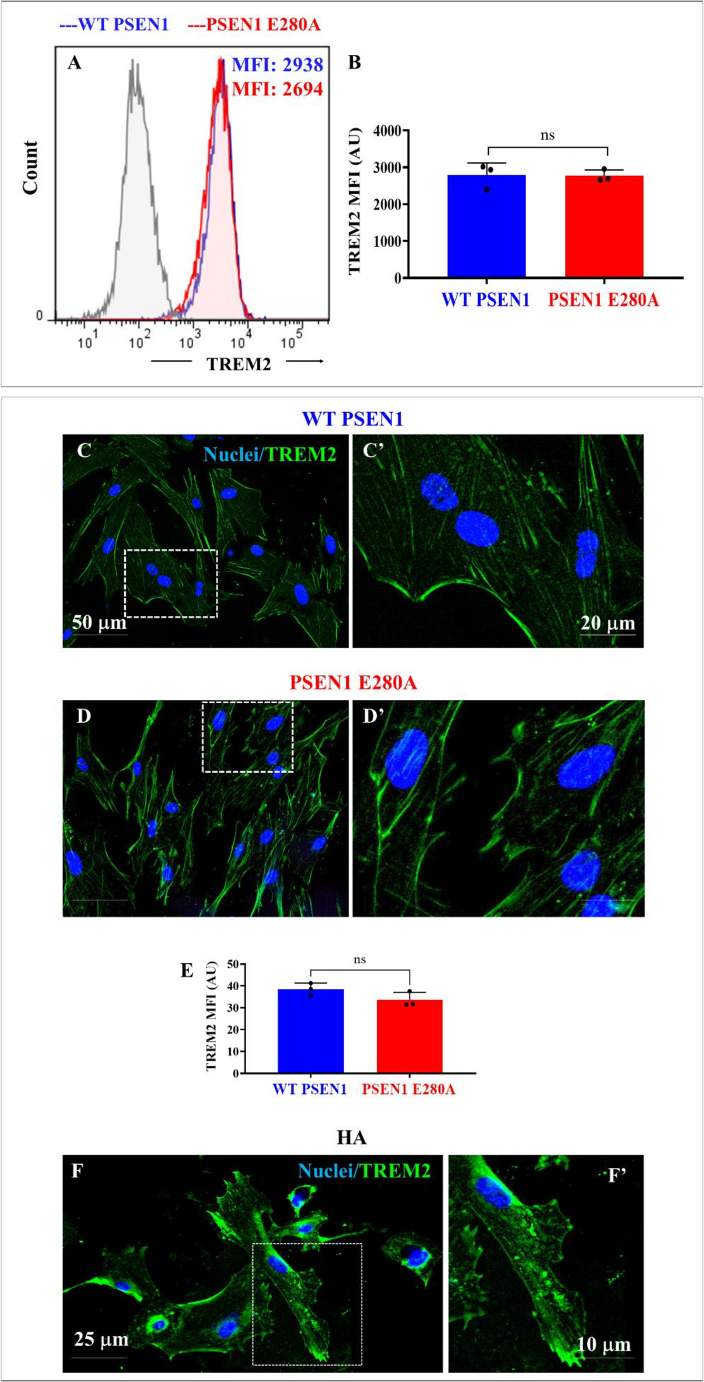
PSEN1 E280A ALCs exhibit similar TREM2 expression compared to WT cells. Representative flow cytometry histograms showing the mean intensity fluorescence of PSEN1 WT (blue fluorescence) and PSEN1 E280A ALCs (red fluorescence) stained with antibodies against superficial TREM2 (**A**). Quantification of TREM2 as mean fluorescence intensity (**B**). Merge fluorescence microscopy images showing double stained with antibodies against TREM2 (green fluorescence) in WT PSEN1 (C, inset broken white line C’) and PSEN1 E280A (**D**, inset broken white line D’). The nuclei were stained with Hoechst 33,342 (blue fluorescence, **C**-**D**). Quantification of TREM2 mean fluorescence intensity (**E**). Merge fluorescence microscopy images showing double stained with antibodies against TREM2 (green fluorescence) in human astrocytes (**F**, inset broken white line F’). Data are expressed as the mean ± SD; ns = not significance. Image magnification 20x. Inset magnification 100x

The autophagy–lysosomal pathway is known to play a critical role in intracellular clearance and metabolic homeostasis in astrocytes [51]. We therefore aimed to evaluate autophagy in mutant ALCs. The detection of microtubule-associated protein 1 A/1B-light chain 3 (LC3) is key to understanding autophagy. During autophagy, the cytosolic form of LC3 (LC3-I) is conjugated to phosphatidylethanolamine to form LC3-phosphatidylethanolamine (LC3-II), which is then recruited to the membranes of autophagosomes [52]. Therefore, LC3-II is indeed widely used as a marker for autophagosomes, and the quantification of LC3-positive puncta is considered the gold-standard assay for determining the number of autophagosomes in cells. As shown in Fig. 8I, WT ALCs displayed a basal percentage of LC3-II + cells (7%), whereas PSEN1 E280A ALCs exhibited a significant increase of + 543% LC3-II + cells (Fig. 7I) compared to WT ALCs (Fig. 7J). These observations were confirmed by immunofluorescent microscopy analysis (Fig. 7K–M).

Several data have shown that the function of TREM2 (a cell surface receptor widely expressed on astrocytes [53]) in AD is primarily related to the clearance of soluble and insoluble amyloid beta (Aβ42) aggregates [54] and the recognition of apoptotic cells that release phospholipids in dying cells by apoptosis [55]. Accordingly, we investigated whether the expression of total TREM2 in ALCs was altered by the PSEN1 E280A mutation. As shown in Fig. 8, WT ALCs express basal levels of TREM2 protein, but its expression was not affected in mutant ALCs (Figs. 8A-B). Similar observations were obtained by immunofluorescence microscopy analysis (Figs. 8D-F).

### Astrocyte-like cells (ALCs) carrying PSEN1 E280A mutation activate pro-inflammatory NF-κB and secrete high extracellular leve of IL-6

**Fig. 9:**
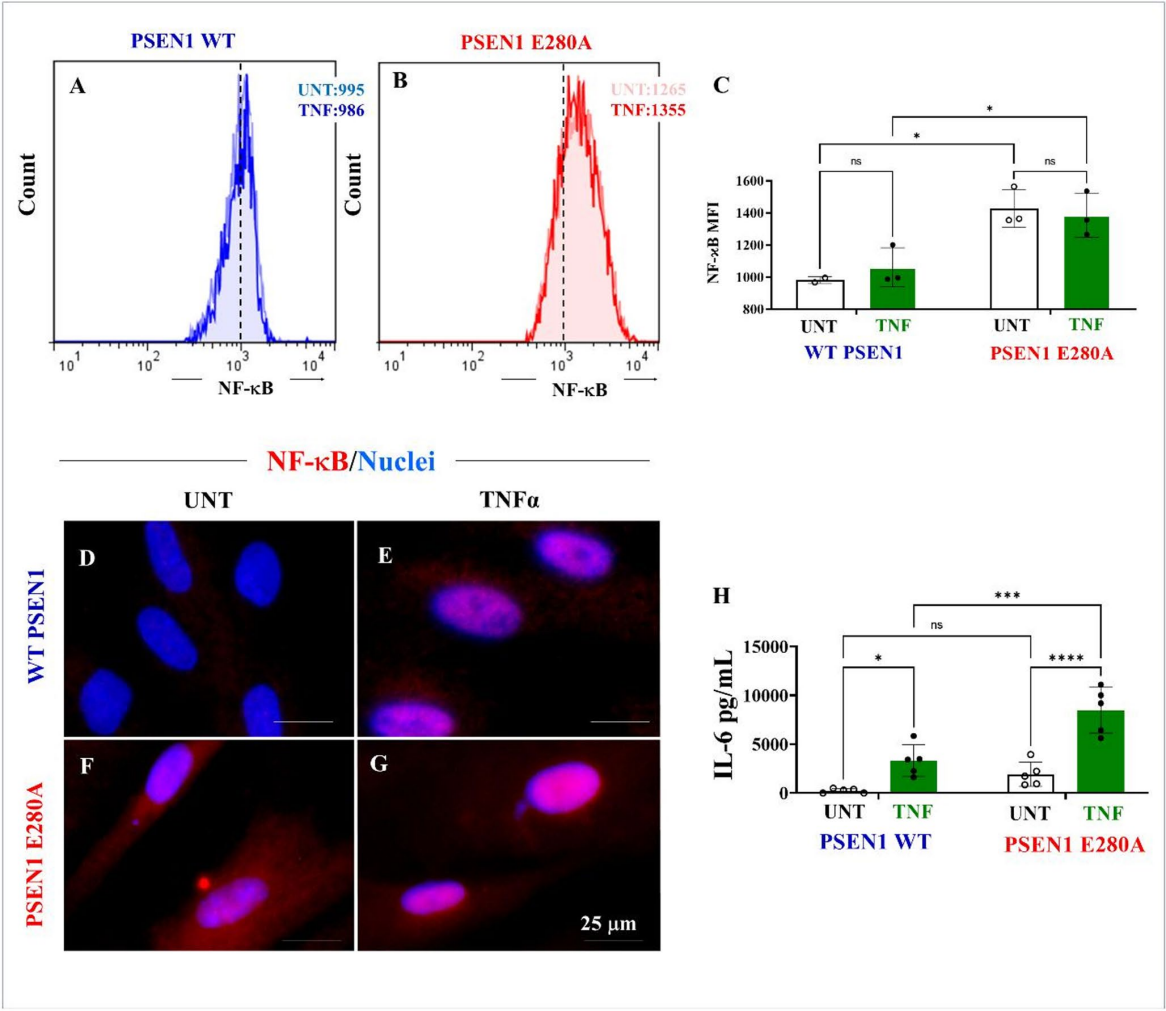
PSEN1 E280A ALCs endogenously activates NF-κB and secrete high levels of NF-κB-induced IL-6. WT and PSEN1 E280A ALCs were either left in regular culture medium or exposed to TNFα for 24 h. Representative flow cytometry histograms showing the fluorescence intensity of NF-κB positive WT (**A**) and PSEN1 E280A (**B**) ALCs. NF-κB expression as mean fluorescence intensity (**C**). Merge fluorescence microscopy images showing stained with antibodies against NF-κB in untreated WT (**D**) or untreated PSEN1 E280A ALCs (**E**) and in WT (**F**) or PSEN1 E280A ALCs (**G**) treated with TNFα. The nuclei were stained with Hoechst 33,342 (blue fluorescence, **D**-**G**). ALCs supernatants were collected and used to determine IL-6 levels as described in the Materials and Methods section. Quantification of IL-6 (**H**). Data are expressed as the mean ± SD; *p < 0.05; ***p < 0.001; **** p < 0.0001; ns = not significance. Image magnification 100x. Inset magnification 100x

To further characterize the immunological response of astrocytes, ALCs were exposed to the inflammatory cytokine TNF-α to evaluate activation of NF-κB (a pro-inflammatory transcription factor induced by TNF-α [56]), and secretion of IL-6 (an interleukin induced by NF-κB [57]),. Figure 9 shows that untreated PSEN1 E280A tended to endogenously activate NF-κB by + 27% compared to untreated ALCs (Figs. 9A-C). Under TNF-α exposure, PSEN1 E280A moderately increased the activation of NF-κB by + 37% compared to untreated ALCs (Figs. 9A-C). Notably, untreated WT ALCs showed low reactivity for nuclear or cytosolic NF-κB (Fig. 9D), but upon exposure to TNF-α, these cells showed nuclear detection of NF-κB-positive cells by immunofluorescence microscopy (Fig. 9E). Surprisingly, untreated PSEN1 E280A ALCs exhibited both cytosolic and nuclear NF-κB-positive cells (Fig. 9F), but under TNF-α the detection was even more pronounced at the nuclear localization (Fig. 9G). Analysis of secreted IL-6 by cytometric bead arrays shows that while untreated WT ALCs secreted low basal levels of IL-6 (238 pg/mL), WT ALCs exposed to TNF-α secreted 3327 pg/mL, a + 1298% increase in IL-6 (Fig. 9H). Interestingly, untreated PSEN1 E280A ALCs secreted a high basal level of IL-6 (1935 pg/mL, + 713% increase) compared to untreated WT ALCs, whereas mutant ALCs treated with TNF-α increased IL-6 secretion by + 338% (8478 pg/mL IL-6) compared to untreated PSEN1 E280A ALCs (Fig. 9H).

## Discussion

Previously, MenSCs derived from menstrual blood have been shown to be a cost-effective, suitable, and reliable source for obtaining ALCs in 7 days [37] compared to other sources, e.g., fibroblast-derived iPSC-derived astrocytes [33], which require at least 98 days to obtain human astroglia in vitro. We confirm that MenSCs can differentiate into mesodermal lineage osteocytes, adipocytes, and chondrocytes, comparable to MSCs derived from other tissue sources [36, 58]. Indeed, the ability to differentiate into mesodermal lineages was not affected or diminished in PSEN1 E280A MenSCs. These observations suggest that the PSEN1 E280A mutation does not affect the cellular differentiation process of MenSCs. In addition, both WT and mutant MenSCs (day 0) expressed basal protein levels of astrocyte lineage markers such as GFAP and S100β. Therefore, MenSCs appear to be primed to express glial markers at early stages of development. Consistent with [38], MenSCs can transdifferentiate into ALCs. Interestingly, while the expression of GFAP was almost similar in WT and mutant MenSCs and WT ALCs, mutant MenSCs expressed higher S100β than WT MenSCs, but these levels remained similar after ALC transdifferentiation. The biological significance of the increased expression of S100β in MenSCs and in the pathophysiology of AD, particularly in astrocytes, remains to be fully elucidated [59].

Although PSEN1 E280A did not alter the basal phenotypic profile and differentiation properties of MenSCs, we observed that mutant ALCs failed to respond to Glu-induced Ca^2+^ influx, APP processing, autophagy-lysosomal activity, and phagocytosis in a manner that was both dependent and independent of intracellular Aβ accumulation. Several observations support this notion. First, in agreement with others [60], where human iPSC-derived astrocytes with PSEN1 exon 9 deletion exhibited increased intracellular production of Aβ, we report for the first time that PSEN1 E280A ALCs generated intracellular accumulation of Aβ peptide. Therefore, intracellular accumulation of Aβ may be a marker not only for FAD astrocytes (this work [26]), but also for neuronal cells, as demonstrated in postmortem human brains of AD patients [61], rats [62, 63], transgenic mouse models of AD [64–67], and umbilical cord Wharton’s jelly mesenchymal stromal cell-derived cholinergic-like neurons [36]. Second, we found that both markers of apoptosis, CASP3 (i.e., CC3 + cells) and ΔΨ_m_ disruption remained unaffected in PSEN1 E280A ALCs to a similar extent as in WT ALCs. These observations suggest that the accumulation of iAβ, which serves as a signaling trigger that affects the ΔΨ_m_ and activation of CASP3, is insufficient to induce cell death signaling in ALCs. However, the aberrant functionality of ALCs occurs independently of PSEN1 mutation. Third, in agreement with this last statement, we found that PSEN1 E280A ALCs displayed a markedly dysfunctional phagocytosis activity as evaluated by the pH-rodo^®^ *E. coli* fluorescence bioparticles^®^ phagocytosis assay and the engulfment assay of PI-apoptotic bodies derived from leukemic cells treated with TPEN [44]. Interestingly, PSEN1 E280A ALCs showed a −74% decrease in double staining of PI + apoptotic bodies/Lysotracker^®^. One possible explanation is that by disrupting PSEN1-dependent targeting of the v-ATPase V0a1 subunit to lysosomes, KO or mutations in PSEN1 E280A altered substrate proteolysis and phagosome/autophagosome clearance during phagocytosis, which in turn caused a selective impairment of phagolysosome/autophagolysosome acidification [68]. In addition, PSEN1 deficiency may also decrease autophagy in human neural progenitor cells via downregulation of ERK/CREB signaling [69]. To support these observations, we found that PSEN1 E280A ALCs displayed high levels of LC3-II, indicating an abnormal accumulation of autophagosomes. In agreement with others [70], our findings suggest that LC3-II-positive structures are prominent in autophagy-deficient PSEN1 E280A ALCs. Taken together, these observations therefore suggest that impaired acidification of lysosomes, accumulation of autophagosome, and alterations in the formation of autolysosome/autophagolysosome [71] may contribute to the inefficient process of engulfment, and clearance of apoptotic bodies, iAβ42 and eAβ42. Alternatively, the lack of engulfment of PI + apoptotic bodies and the slow degradation in autophagolysosomes are consistent with the notion of reduced phagocytic capacity and autophagy-lysosomal pathway dysfunction in astrocytes from FAD [72]. Interestingly, a similar conclusion was reached by [73], who showed that eAβ directly impairs the ability of astrocytes to clear the pathological accumulation of oligomeric Aβ in primary astrocyte cultures to phagocytose and degrade isolated synapses (synaptoneurosomes) from APP (containing dystrophic synapses and Aβ peptides) mice. Finally, mutant ALCs did not respond to Glu-induced Ca²⁺ influx. Although the exact mechanism by which PSEN1 mutations, particularly E280A, affect calcium influx activity in ALCs is not yet known, PSEN1 mutations may affect metabotropic glutamate receptors (mGluRs) [74]. Since PSEN1 E280A ALCs secrete significant amounts of Aβ42, it is possible that Aβ42 may directly interact with mGluRs [75], thereby affecting the response to glutamate-induced calcium influx. However, further investigation is needed to determine exactly which of the mGluRs are impaired in our paradigm model. Taken together, these observations suggest that PSEN1 E280A mainly affects phagocytic capacity, lysosomal activity, autophagy-lysosomal pathway, and Ca^2+^ signaling in PSEN E280A ALCs, thereby contributing to AD progression.

In support of the above, we also found that unstimulated mutant ALCs endogenously express the pro-inflammatory transcription factor NF-κB and secrete abnormal levels of pro-inflammatory cytokines such as IL-6. In addition, the mutant ALCs were much more responsive to pro-inflammatory stimuli, such as exposure to TNFα, than WT ALCs. These observations suggest that mutant ALCs are primed to secrete inflammatory molecules, thereby contributing to an enhanced neuroinflammatory cerebral environment that may alter astrocyte-neuron signaling and/or communication in AD [76]. Interestingly, TREM2, a highly expressed receptor in microglia [77], is also expressed in astrocytes. We found that mutant ALCs expressed similar levels of TREM2 as WT ALCs. Since TREM2 expression is associated with the rate of phagocytosis [78], this may explain why PSEN1 E280A ALCs exhibited low phagocytosis, e.g., in the apoptotic body assay (this work). Given that PSEN1 regulates intracellular TREM2 trafficking [79] and proteolytic processing of TREM2 [80], it is highly possible that mutations in PSEN1, such as the E280A, could alter the intracellular TREM2 location in the plasma membrane, which in turn induces a dysfunction in the phagocytosis process in ALCs. Indeed, it has been shown that hiPSCs-derived microglia-like cells harboring TREM2 missense mutations exhibit specific deficits in phagocytosis [81]. Taken together, these results suggest that the PSEN1 E280A mutation in ALCs promotes a trend towards a more pro-inflammatory profile, but with a significant reduction in, e.g., Aβ clearance capacity via abnormal phagocytic processes and/or deficient autophagy-lysosomal clearance. Therefore, high secretion of pro-inflammatory cytokines (e.g., IL-6) and deficient clearance of Aβ by PSEN1 E280A ALCs may be critical in exacerbating the etiopathogenesis of FAD, such as the accumulation of Aβ42 in plaques and exacerbated neuroinflammatory processes in AD brains.

Our study had several strengths. The WT MenSCs were able to generate ALCs in 7 days when cultured in a defined Astrocyte Medium^®^ and retained the expression of markers consistent with mature astrocytes, such as GFAP and S100β. Functionally, WT ALCs responded to Glu-induced Ca^2+^ influx, secreted pro-inflammatory cytokines (e.g., IL-6), increased NF-κB reactivity/nuclear localization when challenged with cytokines (e.g., TNF-α), expressed the TREM2 receptor, secreted insignificant amounts of Aβ42, maintained a balanced in the autophagy lysosomal pathway markers (lysosomal and autophagosome), and maintained normal phagocytic activity toward cellular debris (e.g., apoptotic bodies). These characteristics were highly altered in the PSEN1 E280A mutation. Consistent with our findings regarding impaired autophagy in MenSCs-derived E280A ALCs, Almeida et al. [82] analyzed PSEN1 E280A in post-mortem frontal cortex tissue using single-nucleus RNA sequencing (snRNA-seq). They demonstrated that several genes associated with autophagy were overexpressed in PSEN1 E280A astrocytes, including HSP90AB1, HSPA9, and ATG8 family members (GABARAPL2 and GABARAPL1). The authors also found overexpressed genes associated with mitochondrial complexes I, III, IV, and V. It is unknown whether the overexpressed genes are associated with a decrease or increase in mitochondrial respiration function. However, we found that mitochondrial potential remains unaltered PSEN1 E280A ALCs. Our study also had limitations. Some of the observed differences between WT and E280A ALCs may be due to inter-individual variability, as cells from genetically different donors were used (e.g., different APOE profile). Therefore, the conclusions drawn from our present investigation need to be verified by additional studies with larger sample sizes of WT. Although ALCs shared the polygonal to fusiform and flat morphology of proliferative astrocytes under basal conditions [83, 84], under our culture conditions they did not show the typical astrocytic morphology of some astrocyte populations characterized by round cells with numerous fine processes extending in all directions, i.e., star-like cells [18]. Neuroinflammation-induced reactive astrocytes, known as type A1 cells, normally exhibit cell hypertrophy, elongation, process extension, and arborization. However, under the present conditions, e.g., the TNFα-induced reactive ALCs with no significant morphological changes. Consistent with the idea that astrocytes are a class of neural cells (astrocytes) of neuroectodermal origin that show highly heterogeneous morphology [85], our results suggest that under the present experimental conditions, ALCs resemble mature astrocytes, with a large proportion showing a fibroblast-like cell morphology. Lastly, antibody 1E8 does not exclusively recognize Aβ42. Given that astrocytes are known to produce Aβ40 as well as Aβ42, the detection of Aβ42 may be misleading. However, due to the prevalence of γ-secretase/PSEN1 E280A in generating Aβ42 over Aβ40, under the present experimental conditions, antibody 1E8 mostly identifies Aβ42 fragments.

## Conclusions

**Fig. 10:**
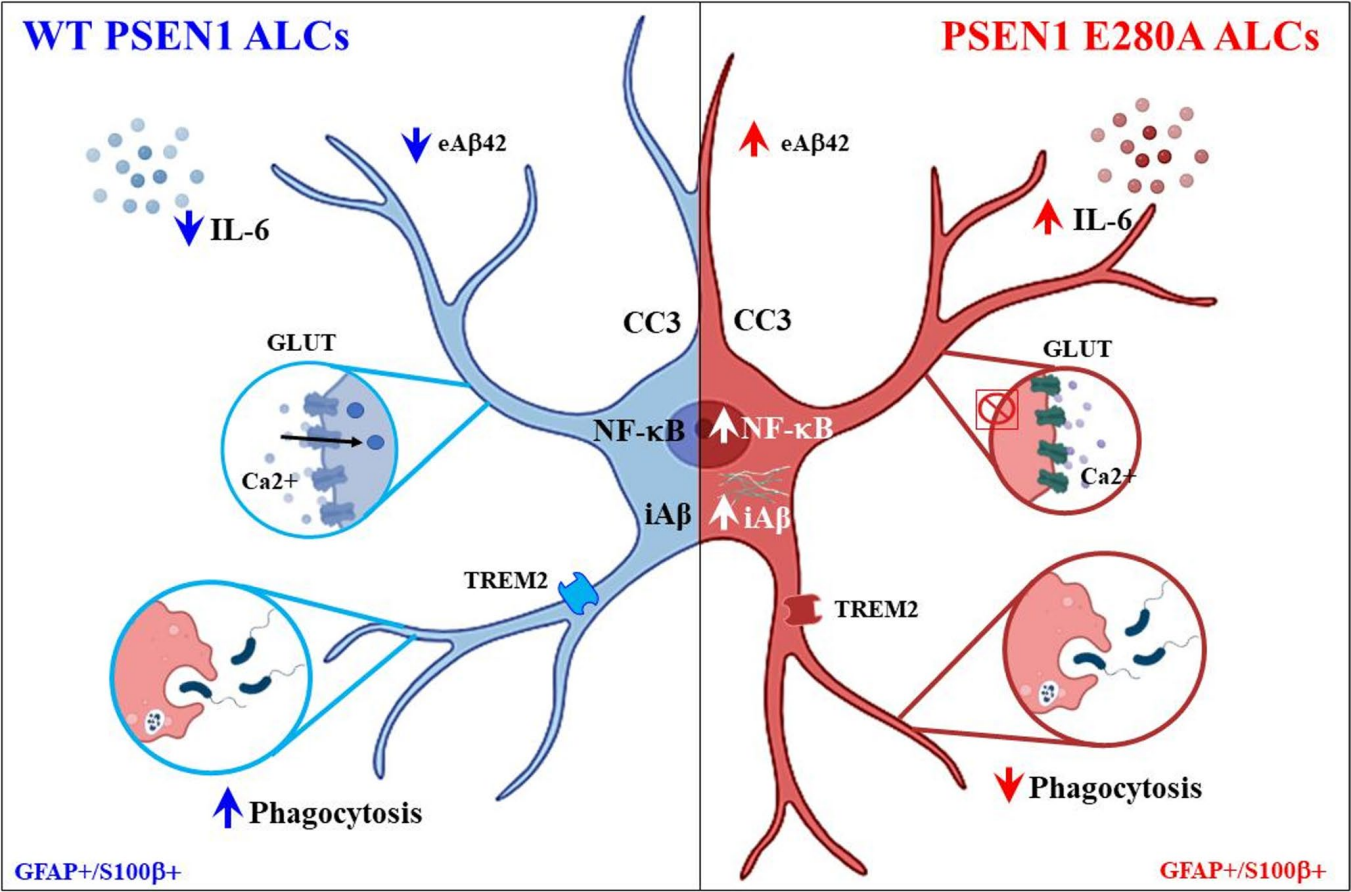
Schematic representation of the main features displayed by wild type (WT) astrocyte-like cells (ALCs) versus PSEN1 E280A ALCs derived from menstrual stromal cells (MenSCs)

In vitro models of human astrocytes (e.g., embryonic stem cells, directly reprogrammed fibroblasts, immortalized cells, and iPSC-derived astrocytes) are time-consuming and expensive to obtain under experimental laboratory conditions [86–88]. We have obtained ALCs derived from WT and PSEN1 E280A MenSCs that require only 7 days for their transdifferentiation. Indeed, the MenSCs-derived ALCs displayed several features of mature astrocytes, including expression of the typical astrocytic markers GFAP+/S100β + and TREM2 receptor, and were able to recapitulate several physiological functions, including inflammatory (e.g., secreted IL-6, NF-κB-positive cells), calcium responses to stimuli (e.g., Glu-induced Ca^2+^ influx) and phagocytic activity. We also showed that the PSEN1 E280A mutation impaired phagocytic activity and abolished the Glu-induced Ca^2+^ influx response (Fig. 10). Our results suggest that mutant ALCs in FAD may be involved in an ineffective clearance process of Aβ1-42 protein, thereby contributing to the uploading and accumulation of Aβ into amyloid plaques and creating a more pro-inflammatory environment, which seriously contributes to the pathophysiology of FAD. The development of this model provided a unique opportunity to better understand the cellular interplay in the brains of FAD patients, as well as the influence of cell-specific effects of the FAD-associated PSEN1 E280A mutation and the potential interactions between astrocytes and neurons carrying the PSEN1 E280A mutation.

## Data Availability

All relevant data are within the manuscript.

## Declarations

### Permission to reproduce material from other sources

Not Apply.

### Clinical trial registration

Not Apply.
